# Regional patterns of declining butternut (*Juglans cinerea* L.) suggest site characteristics for restoration

**DOI:** 10.1002/ece3.3641

**Published:** 2017-12-01

**Authors:** Randall S. Morin, Kurt W. Gottschalk, Michael E. Ostry, Andrew M. Liebhold

**Affiliations:** ^1^ USDA Forest Service Northern Research Station Newtown Square PA USA; ^2^ USDA Forest Service Northern Research Station Morgantown WV USA; ^3^ USDA Forest Service Northern Research Station St. Paul MN USA

**Keywords:** butternut, *Juglans cinerea*, *Ophiognomonia clavigignenti‐juglandacearum*, plant disease, restoration, species distributions

## Abstract

Butternut trees dying from a canker disease were first reported in southwestern Wisconsin in 1967. Since then, the disease has caused extensive mortality of butternut throughout its North American range. The objectives of this study were to quantify changes in butternut populations and density across its range and identify habitat characteristics of sites where butternut is surviving in order to locate regions for potential butternut restoration. The natural range of butternut (*Juglans cinerea* L.) extends over a large region of eastern N. America encompassing New Brunswick south to North Carolina, north to Minnesota, and southwest to Missouri. Despite the species’ large range, it is typically not a common tree, comprising a relatively minor component of several different forest types. We evaluated change in butternut abundance and volume from current and historic data from 21 states in the eastern United States. We related abundance and volume at two time periods to a suite of ecological and site factors in order to characterize site conditions where butternut survived. We also assessed the current level of butternut mortality across its range. Since the 1980s, the number of butternut trees and butternut volume have decreased by 58% and 44%, respectively, across its US range. Substantial relative decreases in tree numbers and volume occurred in most ecoregion sections. Five environmental variables were found to be significant predictors of butternut presence. The potential impacts of butternut canker are particularly acute as the canker pathogen invasion pushes a rare tree species toward extinction, at least at a local scale. Based on the results presented here, large‐diameter maple/beech/birch stands in dry, upland sites in eastern Minnesota, western Wisconsin, and upstate New York appear to offer the most favorable conditions for butternut growth and survival and thus may be the best stands for planting resistant butternut trees.

## 1 INTRODUCTION

**Figure 1:**
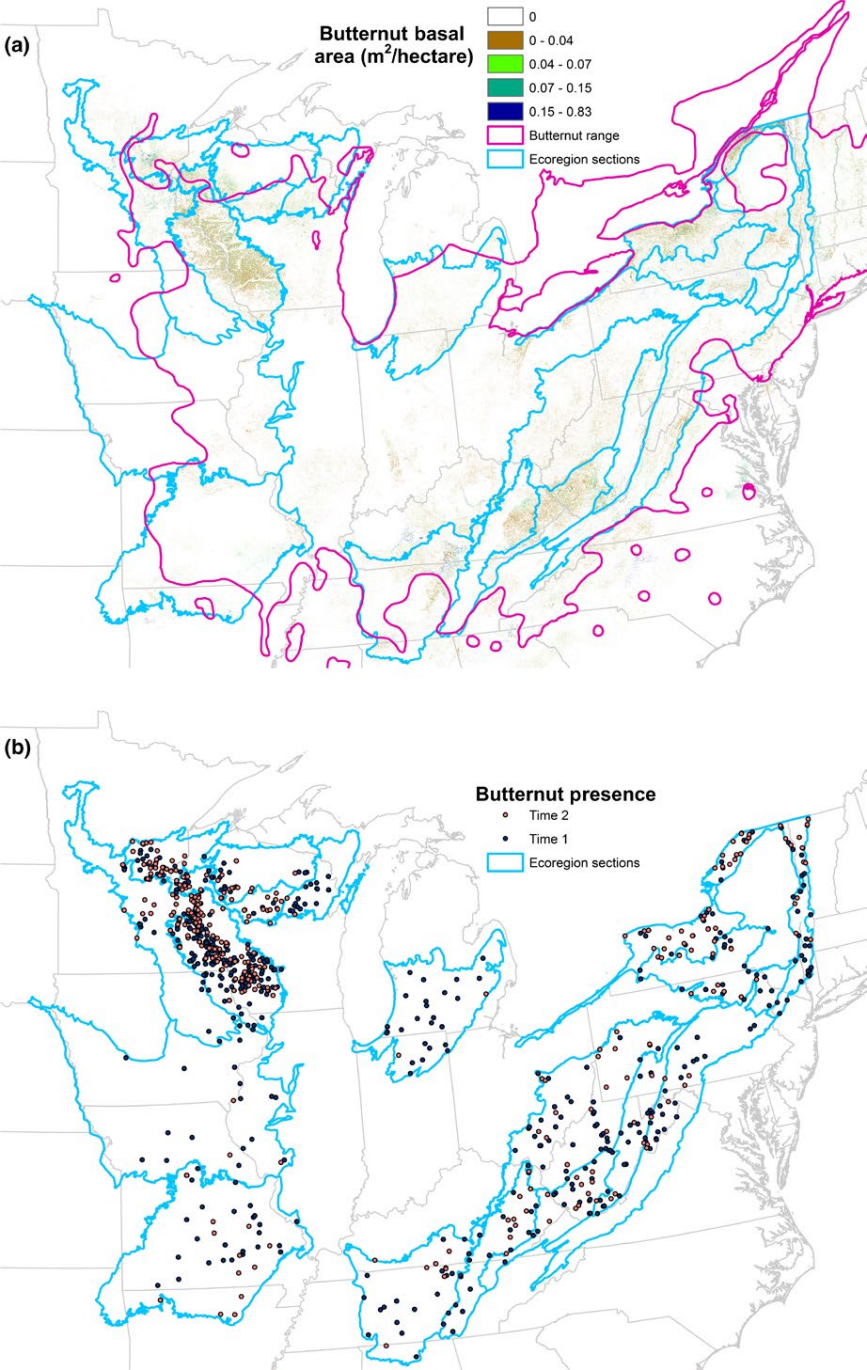
(a) Butternut basal area per acre of forest land (Wilson, Lister, Riemann, & Griffith, 2013), historic range of butternut (Little, 1971), and ecoregion section boundaries in the eastern United States, (b) FIA plot locations with butternut present at time 1 and time 2 (plot locations are approximate)

For over two centuries, North American butternut (*Juglans cinerea* L.) was cherished for its exceptional wood properties and was sought after for the manufacture of fine furniture, musical instruments, and boats (Woeste & Pijut, 2009). The species was also valued for its sweet, oily nuts that were desired by both Native Americans and European settlers and are also a source of large mast utilized by various wildlife species. The natural range of butternut extends over a large region encompassing New Brunswick south to North Carolina, north to Minnesota, and southwest to Missouri. Isolated, scattered butternut also occur in Arkansas, Mississippi, Alabama, Georgia, and South Carolina (Little, 1971; Figure 1a). Despite the species’ large range, it is typically not a common tree, comprising a relatively minor component of several different forest types.

Butternut trees dying from a canker disease were first reported in southwestern Wisconsin in 1967. Since then, extensive mortality of butternut of all ages has been observed throughout its North American range (Ostry, 1998a; Ostry & Woeste, 2004). This disease is caused by the fungal pathogen *Ophiognomonia clavigignenti‐juglandacearum* (syn. *Sirococcus clavigignenti‐juglandacearum*—Broders & Boland, 2011) which is non‐native in North America and possibly introduced from Asia (Furnier, Stolz, Mustaphi, & Ostry, 1999; Nair, 1998; Ostry, 1998b; Ostry, Mielke, & Skilling, 1994). A recently described twig endophyte of *Acer truncatum* in China may also have been a close relative of *O. clavigignenti‐juglandacearum* (Sun, Guo, & Hyde, 2011); if so, it is the only recorded occurrence of the fungus outside of North America. In 2004 the U.S. Forest Service, Forest Health Protection program estimated that 77% percent of the butternut trees in North Carolina and Virginia had been killed, and in the northeastern U.S., most of the monitored butternut trees were affected by butternut canker (U.S. Forest Service, 2005). A variety of different insect species are capable of carrying spores of the pathogen thus possibly explaining its widespread distribution (Halik & Bergdahl, 2002; Katovich & Ostry, 1998), and spores can remain viable for long periods (Moore & Ostry, 2015). The extent of the disease is so great that butternut is increasingly rare and is considered an imperiled species in many states (Woeste & Pijut, 2009). Butternut is listed as a “species of concern” or a “sensitive species” in several states and is a Regional Forester Sensitive Species on 13 of the 16 National Forests in the Eastern Region of the US Forest Service. Butternut is listed as endangered in Canada.

Initial genetic analysis of *O. clavigignenti‐juglandacearum* from several North American locations indicated that it may have been introduced into North America as a single strain (Furnier et al., 1999). Recent evidence supports an introduction of three genetic clusters of the fungus and only asexual reproduction (Broders & Boland, 2011). This finding provides some optimism that resistance in butternut might eventually be found and may not be quickly overcome by the development of increasingly pathogenic races of the pathogen.

Several studies have evaluated the genetic structure and diversity of butternut with results than can guide sound management of a declining species. For example, a study by Ross‐Davis, Ostry, and Woeste (2008) reported that butternut retains a large amount of genetic diversity that is higher than previously estimated, and Boraks and Broders (2016) found significant gene flow among butternut populations in the northeast. Parks, Jenkins, Ostry, Zhao, and Woeste (2014) also found that genetic diversity in 19 watersheds in the Great Smoky Mountains National Park was evenly distributed with high mean heterozygosity although there was variability in some subpopulations due to hybridization with Japanese walnut (*Juglans ailantifolia*). Hybridization has been reported to occur across a large portion of the range of butternut (Hoban, McCleary, Schlarbaum, & Romero‐Severson, 2009), and hybrids are virtually indistinguishable from true butternut, so it is possible that some trees recorded during the forest inventories reported here could be hybrids.

There has been good progress in the methods for identifying resistant butternut, collecting germplasm, and hybridizing butternut and Japanese walnut (Michler et al., 2005; Ostry & Moore, 2008; Woeste & Pijut, 2009). Given the progress in identifying potentially resistant trees, careful consideration is being given to the selection of locations where reintroductions are most likely to be successful (Woeste, Farlee, Ostry, McKenna, & Weeks, 2009). A predictive model was developed to identify potential sites for butternut restoration in Mammoth Cave National Park (Thompson, Van Manen, Schlarbaum, & DePoy, 2006), but butternut restoration has not been studied across its historic range.

The characteristics of the forest lands where surviving butternut trees remain can provide guidance for where restoration efforts should be focused. Butternut exhibits its best growth on well‐drained soils and streambanks and is rarely found on infertile, compact, or dry soils. Additionally, the species is most often present in coves, stream benches, slopes, and other sites with good drainage up to elevations of about 1,500 meters. The most common associated tree species include basswood (*Tilia* spp.), black cherry (*Prunus serotina* Ehrh.), American beech (*Fagus grandifolia* Ehrh.), black walnut (*Juglans nigra* L.), eastern hemlock (*Tsuga canadensis* (L.) Carr.), hickory (*Carya* spp.), and oak (*Quercus* spp.). Butternut is considered to be a shade‐intolerant species and cannot tolerate shade from competition above (Burns & Honkala, 1990).

The objectives of this research were to map the occurrence of surviving butternut in the United States using nationwide forest inventory data and quantify changes in butternut populations and density across its range in order to determine the potential impacts of butternut canker. An additional objective was to identify spatial trends in butternut changes and identify ecological characteristics of stands where butternut is surviving in order to suggest the potential characteristics of forests where butternut restoration is most likely to be successful.

## 2 METHODS

The Forest Inventory and Analysis (FIA) program of the U.S. Department of Agriculture, Forest Service, conducts an inventory of forest attributes across the country (Bechtold & Patterson, 2005). The FIA sampling design is based on a tessellation of the United States into hexagons approximately 2,428 ha in size with at least one permanent plot established in each hexagon.

Prior to 1999, FIA collected data regionally using a periodic measurement system with sample designs that varied slightly by region. Generally, inventories were conducted in each state every 6–18 years, depending on the state and region (Bechtold & Patterson, 2005). Beginning in 1999, FIA moved to an annual inventory approach where a complete, systematic sample of each state is completed annually (Bechtold & Patterson, 2005).

Current and historic data from 21 states in the eastern United States were extracted from the FIA database (Woudenberg et al., 2010; DataMart link—https://apps.fs.usda.gov/fia/datamart/datamart.html). Ecoregion section‐level (McNab et al., 2007) and plot‐level estimates of butternut populations (number of live trees ≥2.54 cm d.b.h.) and total butternut volume (m^3^ of live trees ≥2.54 cm d.b.h.) were generated for all states in the historic range of butternut (Little, 1971) at two time periods (Appendix S1) and by diameter class. Due to the variability in timing of the periodic inventories and implementation of annual inventories, the interval of time between surveys differs among states and was averaged for each ecoregion section (Appendix S2). As the periodic and annual inventories are completely independent samples, it is not possible to directly attribute the death of individual trees. Therefore, long‐term (multidecadal) changes in butternut abundance and density were estimated at regional levels instead of for individual trees.

Annualized change was calculated for each state by dividing the difference between estimates at time 1 and time 2 by the number of years between surveys, and relative change was computed by dividing the difference between time 1 and time 2 estimates by the time 1 estimate. In order to test for significant changes in butternut populations and volume across its range and within states, paired t tests were performed with ecoregion section‐ and plot‐level estimates and times 1 and 2 (SAS Institute Inc, 2009) for ecoregion sections that had at least 10 plots containing butternut sampled at time 1. Relative and annualized change in butternut populations and volume were also mapped for all states.

In order to quantify the current level of tree mortality across the geographic range of the study, an annual mortality rate was estimated by state from remeasured annualized FIA data for butternut individually and for all species combined including butternut for comparison. Annual mortality rates were computed by dividing the estimated volume of trees that died between measurement at time 1 and time 2 by the estimated volume of live trees present at time 1 for states with at least 10 remeasured plots containing butternut. These mortality rates are based on the remeasurement period of 2009–2014 for all states except Kentucky, which had a remeasurement period of 2008–2013.

In order to identify ecological characteristics where live butternut trees occur, plots were assigned 0 for butternut absence and 1 for butternut presence, and the probability of butternut occurrence was modeled using logistic regression testing the effects of plot variables that may be good predictors. These variables include ecoregion section (ECOSECT; McNab et al., 2007), ownership group (OWNGRP), stand age (STAGE), physiographic class (PHYSCLCD), forest‐type group (FORTPGRP), and stand‐size class (STDSZCD) (SAS Institute Inc, 2009). ECOSECT and PHYSCLCD are likely to be good indicators of soil types and slope position while OWNGRP, STAGE, FORTYPGRP, and STDSZCD are likely to separate stands by recent management activity and site conditions related to competition, regeneration, and species composition. All included variables were categorical other than STAGE which was continuous. Before logistic models were run, ordinary linear regression, including tolerance and variance inflation diagnostics, was used to test for multicollinearity. None was observed because tolerance was above 0.4 in all cases (Allison, 1999).

Several other tree‐level and stand‐level attributes (elevation, slope, basal area, site index, and aspect) were included initially, but none were found to be statistically significant predictors (α = 0.05), so they were dropped from the models. Odds ratios were evaluated against a reference condition for each categorical variable where the reference condition was assigned as the category with the proportion of plots with butternut presence was closest to the average for the study area. Model goodness of fit was evaluated with the area under the receiver operating characteristic (ROC) curve, max‐rescaled R‐square, and percent accuracy of occurrence classification. The area under the ROC curve is provided for the classification models as an indicator of classification accuracy. An ROC value of 0.5 occurs when the classification is no better than random prediction; a value of 1.0 indicates perfect classification accuracy. A rough guide to interpretation is given by Fischer, Bachman, and Jaeschke (2003): ROC area greater than 0.9 ≈ high accuracy; 0.7–0.9 ≈ moderate accuracy; 0.5–0.7 ≈ low accuracy.

## 3 RESULTS

There is a strong spatial coincidence between Little's (1971) range map and the current distribution of butternut basal area across the eastern United States (Figure 1a). The species occurs at the highest densities in ecological sections 222L, 221H, 221E, and 222I (Figure 1a, Table 1). Although live butternut was sampled in 27 states, it was only sampled on 10 or more plots in 19 ecoregion sections that are comprised of parts of 21 states (Tables 1 and 2). Comparison of plot locations where butternut was present at time 1 versus time 2 indicates an apparent reduction in the geographic distribution over the period from the 1980s through 2015 (Figure 1b).

**Table 1:** Estimates of numbers of butternut trees per hectare of timberland for times 1 and 2 with associated sampling errors, sample size (nonzero plots), annualized change, and relative change by ecoregion section

Ecoregion section	Time 1				Time 2				Annualized change (trees/ha)	Annualized change (trees)	Relative change (%)
	Trees per hectare	Sampling error (%)	Trees	*n* Plots	Trees per hectare	Sampling error (%)	Trees	*n* Plots			
211E	0.899	44	594,997	11	0.947	35	695,324	12	0.00179	3,786	5.3
211F	0.342	30	811,070	17	0.145	47	357,049	6	−0.00856	−19,740	−57.5
212K	0.472	25	485,651	25	0.438	29	486,706	16	−0.00097	30	−7.2
212Q	1.278	63	639,149	15	0.600	35	358,228	11	−0.01936	−8,026	−53.0
212T	0.178	29	230,590	15	0.015	98	20,145	1	−0.00486	−6,282	−91.5
212X	0.148	33	305,718	15	0.095	53	215,605	4	−0.00159	−2,690	−35.9
221B	1.249	38	785,456	14	0.393	71	238,210	3	−0.03260	−20,847	−68.5
221E	0.179	24	920,403	27	0.085	31	438,247	10	−0.00382	−19,680	−52.3
221H	0.269	40	641,880	12	0.123	40	287,386	7	−0.00502	−12,224	−54.2
222I	0.526	47	463,070	11	0.588	29	544,224	11	0.00270	3,528	11.8
222J	0.803	30	793,350	19	0.032	101	44,556	1	−0.02636	−25,600	−96.0
222L	3.970	16	5,230,161	152	0.810	15	1,236,585	49	−0.10113	−127,794	−79.6
222M	1.280	43	543,056	17	0.401	39	261,713	9	−0.02775	−8,885	−68.7
223A	0.085	34	444,555	14	0.046	41	267,948	7	−0.00124	−5,697	−45.6
223E	0.271	38	670,677	10	0.107	47	287,251	6	−0.00538	−12,640	−60.4
251C	0.157	40	272,237	10	0.051	57	114,396	3	−0.00393	−5,846	−67.3
M221A	0.414	25	1,593,290	26	0.244	37	954,127	10	−0.00607	−22,827	−41.0
M221B	0.271	31	533,728	20	0.265	81	514,505	2	−0.00019	−614	−2.2
M221C	0.223	30	529,833	17	0.176	33	433,217	9	−0.00169	−3,420	−21.3
Total	0.182	7	22,389,007	572	0.077	9	9,921,734	222	−0.00363	−430,613	−57.6

**Table 2:** Estimates of the volume per hectare of timberland of butternut trees for times 1 and 2 with associated sampling errors, sample size (nonzero plots), annualized change, and relative change by ecoregion section

Ecoregion section	Time 1				Time 2				Annualized change (vol/ha)	Annualized change (volume)	Relative change (%)
	Volume per hectare	Sampling error (%)	Volume (m^3^)	*n* Plots	Volume per hectare	Sampling error (%)	Volume (m^3^)	*n* Plots			
211E	0.513	42	339,323	11	0.618	39	453,639	12	0.004	4,314	20.4
211F	0.106	31	251,684	17	0.083	51	204,749	6	−0.001	−2,041	−21.5
212K	0.097	23	99,502	25	0.158	35	175,547	16	0.002	2,173	63.4
212Q	0.315	43	157,226	15	0.137	37	81,608	11	−0.005	−2,161	−56.5
212T	0.096	30	124,711	15	0.003	98	3,560	1	−0.003	−3,616	−97.2
212X	0.045	31	91,947	15	0.042	53	95,709	4	0.000	112	−5.4
221B	0.193	36	121,570	13	0.090	81	54,722	3	−0.004	−2,547	−53.3
221E	0.045	22	231,886	26	0.020	41	100,681	10	−0.001	−5,355	−56.5
221H	0.034	39	82,374	12	0.081	56	190,221	7	0.002	3,719	136.2
222I	0.209	52	184,090	10	0.429	36	396,596	11	0.010	9,239	104.9
222J	0.236	26	232,618	19	0.002	101	2,524	1	−0.008	−7,866	−99.2
222L	1.045	11	1,374,819	152	0.190	22	289,629	49	−0.027	−34,726	−81.8
222M	0.313	39	132,588	17	0.110	39	71,570	9	−0.006	−1,927	−64.9
223A	0.016	35	85,207	14	0.008	44	44,260	7	0.000	−1,321	−53.2
223E	0.028	34	70,055	10	0.045	50	119,952	6	0.001	1,645	58.4
251C	0.041	34	71,500	10	0.014	82	31,218	3	−0.001	−1,492	−66.1
M221A	0.079	23	305,147	26	0.077	47	299,493	10	0.000	−202	−3.3
M221B	0.091	29	178,484	20	0.043	77	83,530	2	−0.002	−3,030	−52.5
M221C	0.067	28	158,010	16	0.041	45	100,650	9	−0.001	−2,030	−38.7
Total	0.050	6	6,149,849	568	0.028	11	3,619,843	222	−0.001	−87,385	−43.6

Across its range, butternut populations decreased 58% (*t* = −4.52, *p* < .0001) from 22 million trees (0.07 trees per acre) at time 1 to 10 million trees (0.03 trees per acre) at time 2 (Table 1), and butternut volume decreased 44% (*t* = −2.08, *p* = .0379) from 6.1 million cubic meters (0.05 cubic meters per hectare) at time 1 to 3.6 million cubic meters (0.03 cubic meters per hectare) at time 2 (Table 2). Mean annual change among all ecoregion sections was approximately −431,000 trees per year (0.2 per hectare; Table 1) and −87 million cubic meters of volume per year (0.001 cubic meters per hectare; Table 2). Substantial relative decreases in tree numbers and volume occurred in most ecoregion sections (Tables 1 and 2).

**Figure 2:**
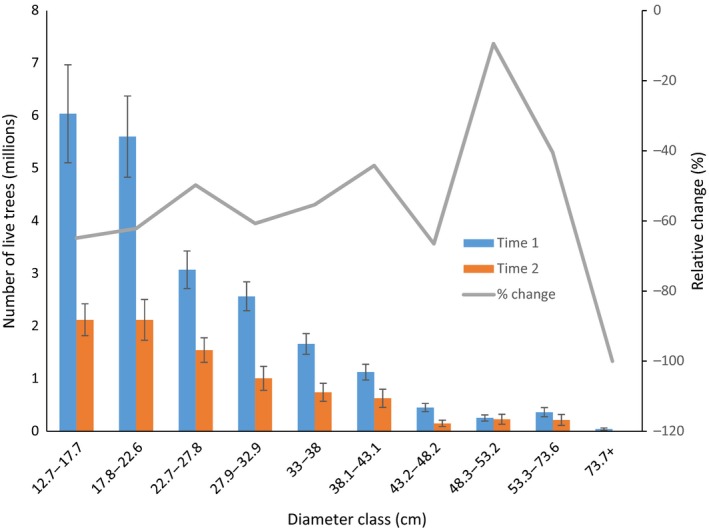
Number of butternut trees by diameter class, time 1 and time 2 and the relative change in numbers of butternut trees over the measurement period

Three of the 19 ecoregion sections that were tested exhibited significant declines in butternut tree populations and volume. The largest significant decreases in butternut populations and volumes, computed as the difference in plot means between time 1 and time 2, were observed in ecoregion sections 212T, 222J, and 222L (Table 3). These ecoregions are located in southeastern Minnesota, northeastern Iowa, western and northern Wisconsin, and southern Michigan (Figure 2). Although increases in butternut numbers and volumes occurred in several ecoregion sections, no significant increases in tree populations or volume were observed for sections that were tested (Table 3). The distribution of butternut trees by diameter class has not changed substantially because populations have decreased by approximately 45%–65% in nearly all classes (Figure 2). Although they made up a small proportion of total butternut trees, two of the four largest diameter classes have the largest relative decreases of all classes.

**Table 3:** Mean difference between time 1 and time 2, sample size, *t* statistic, and *p*‐value for number of trees and volume for the eastern United States and by ecoregion section for butternut

Ecoregion sections	Number of trees				Volume			
	*df*	Mean of difference	*t*	*p*	*df*	Mean of difference	*t*	*p*
All Combined	42,824	−298.1	−4.52	**<.0001**	40,700	−1,531.0	−2.08	**.0379**
211E	512	−154.7	−0.13	.8984	598	2,925.2	0.11	.9132
211F	1,949	−389.6	1.44	.1502	1,900	−987.6	−0.23	.8196
212K	1,605	155.4	0.9	.3697	1,103	3,842.6	1.64	.1014
212Q	656	−503.7	−0.65	.5152	763	−4,800.9	−0.99	.3223
212T	2,358	−128.8	−2.7	**.0071**	1,777	−2,648.1	−3.24	**.0012**
212X	3,671	−17.7	−0.25	.8048	2,754	365.7	0.36	.7204
221B	424	−2,014.9	−1.67	.0958	570	−8,874.8	−1.19	.2362
221E	4,406	−160.2	−1.44	.1511	4,806	−1,580.5	−1.53	.1214
221H	1,289	−379.4	−1.3	.1951	1,266	3,382.4	0.9	.3697
222I	818	311.8	0.47	.6353	712	18,600.1	1.22	.2225
222J	725	−1,093.7	−2.97	**.0031**	688	−11,841.6	−3.5	**.0005**
222L	1,451	−3,057.4	−4.68	**<.0001**	1,683	−29,284.4	−6.25	**<.0001**
222M	811	−483.8	−1.14	.2542	914	−3,765.9	−1.09	.274
223A	7,113	0.2	0	.9976	6,997	−41.5	−0.12	.9074
223E	1,425	−421.9	−1.62	.105	1,929	848.8	0.48	.6336
251C	2,416	−98.5	−1.01	.3102	2,489	−877.9	−0.85	.3953
M221A	2,117	−363.2	−0.87	.3838	2,117	−86.8	0.02	.9842
M221B	1,089	75.9	0.14	.8883	1,805	−2,657.2	−0.81	.4195
M221C	1,723	−45.0	−0.19	.8507	1,535	−1,059.6	−0.4	.6859

Bold indicates significance (*p* < 0.05)

**Figure 3:**
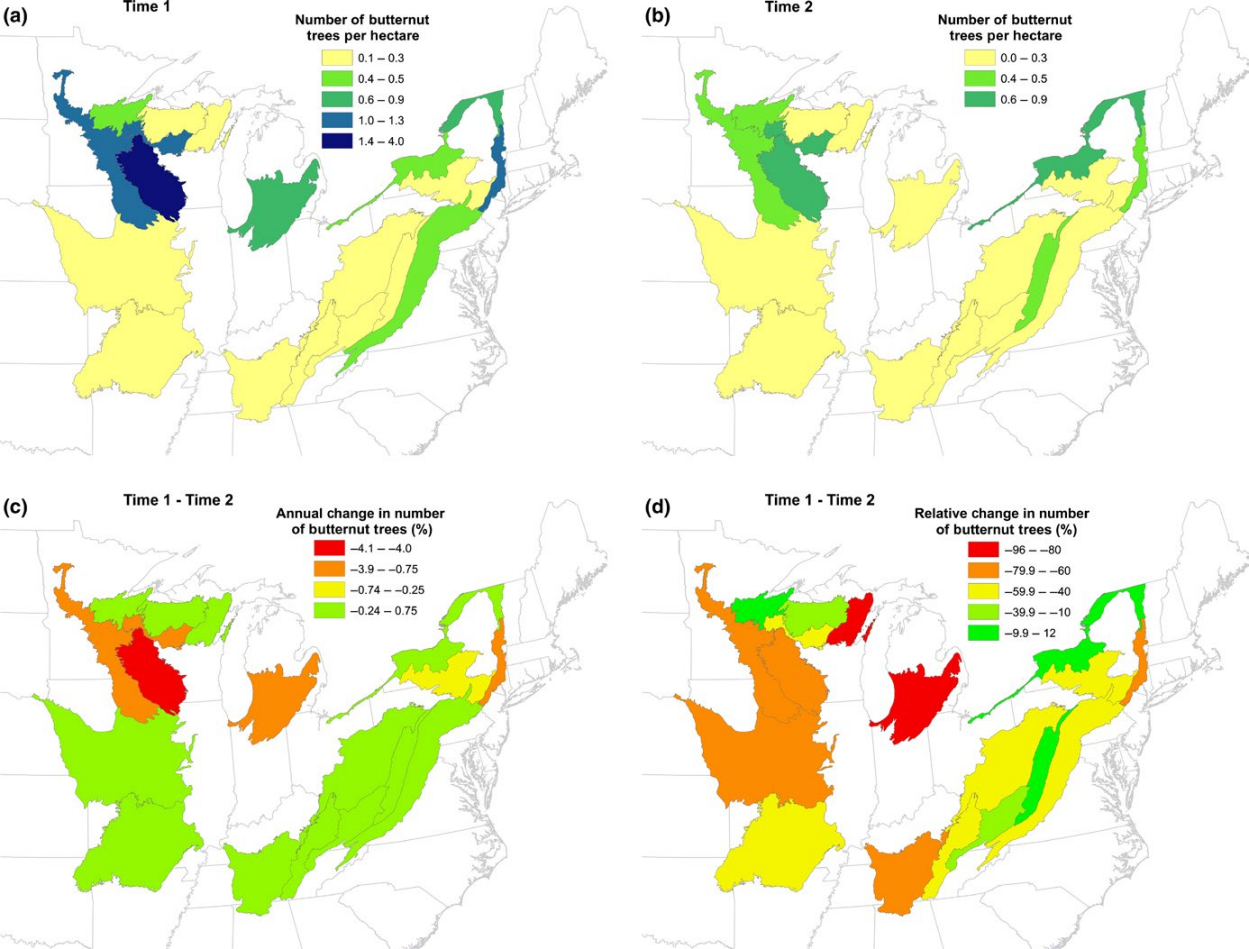
(a) Number of butternut trees per acre, 1980s, (b) number of butternut trees per acre, 2015, (c) annual change in butternut tree numbers, (d) relative change in butternut tree numbers, by ecoregion section for the time interval shown in Table 1

**Figure 4:**
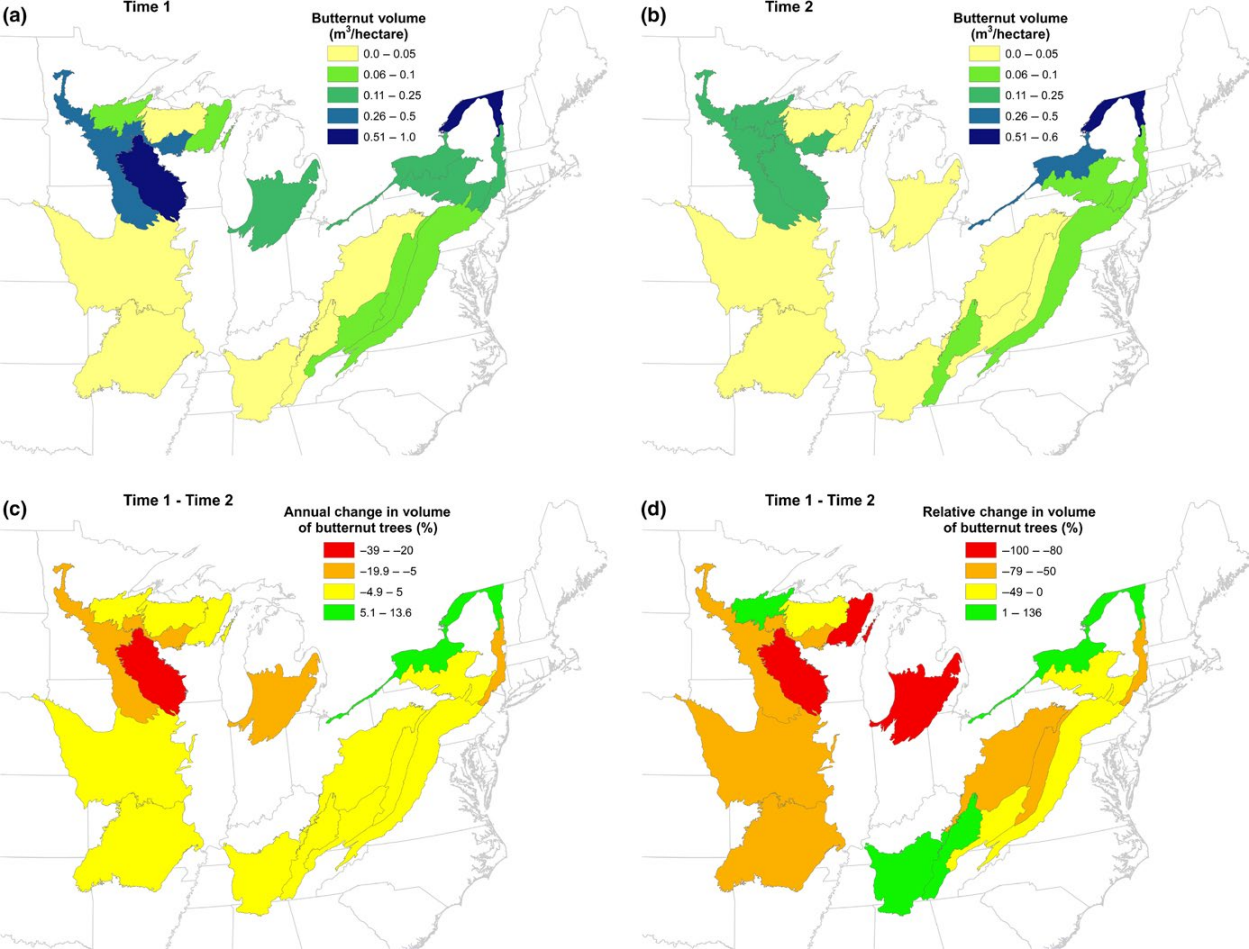
(a) Volume of butternut trees per acre, 1980s, (b) volume of butternut trees per acre, 2015, (c) annual change in butternut volume, (d) relative change in butternut volume, by ecoregion section for the time interval shown in Table 1

The number of live butternut trees and volume decreased in nearly all ecoregions (Tables 1 and 2), but the decreases varied spatially (Figures 3 and 4). The largest annual decreases in numbers and volume occurred in section 222L (Figures 3c and 4c), but the largest relative decreases in numbers occurred in sections 222J and 212T and in volume in sections 222L, 222J, and 212T (Figures 3d and 4d). Although butternut populations decreased in ecoregion sections 223E and 221H, butternut volume actually increased in those sections (Figures 3d and 4d). The annual mortality rate for butternut ranged from about 0.5% in ecoregion sections 221E and 222I to over 10% in 211E and 212K, and the mortality rate for butternut was higher than the rate for all species combined in all sections except 221E and 222I (Table 4).

**Table 4:** Estimates of butternut volume, mortality volume, sample size, and annual mortality rate for ecoregion sections with at least 10 remeasured plots

	Volume of live trees (m^3^)	Volume of mortality trees (m^3^)	*n*	Annual butternut mortality rate^a^	Annual mortality rate^a^ (all species combined)
Total	3,450,990	249,596	204	7.2	1.2
211E	468,569	64,576	12	13.8	1.4
212K	178,104	20,139	15	11.3	1.9
221E	106,279	468	10	0.4	0.9
222I	425,728	2,258	12	0.5	1.1
222L	294,812	24,389	47	8.3	1.5

a Mortality rate was computed by dividing the estimated volume of trees that died between measurement at time 1 and time 2 by the estimated volume of live trees present at time 1 for states with at least 10 remeasured plots containing butternut.

Finally, we found five environmental variables, STAGE, STDSZCD, OWNGRP, FORTPYGRP, and ECOSECT, to be significant (*p* <0 .05) predictors of butternut presence in the original logistic regression models that included all variables for both time periods. A reduced model that included only the significant variables resulted in STAGE no longer being a significant predictor (Table 5) in the model for time 1. To judge the relative importance of the variables, Wald's chi‐square values are given in Table 6. ROC values for the models for both time periods indicated “moderate classification accuracy” (Table 6). The chi‐square statistics in Table 6 reveal that the most important variable for both time periods is ECOSECT. The second, third, and fourth most important variables in both models were FORTYPGRP, STDSZCD, and OWNGRPCD, respectively. STAGE and PHYSCL were the least important variables in both models.

**Table 5:** Estimated parameters for logistic regression occurrence models

Parameter	Time 2					Time 1				
	Odds ratio estimates			Chi‐square	*p*	Odds ratio estimates			Chi‐square	*p*
	Point	Lower	Upper			Point	Lower	Upper		
STAGE	0.991	0.984	0.997	8.1903	**.0042**	1	0.999	1.000	2.0585	.1514
STDSZCD				14.9175	**.0006**				28.2205	**<.0001**
STDSZCD Medium versus Large	1.118	0.838	1.491	0.5723	.4494	0.6890	0.553	0.858	11.0854	**.0009**
STDSZCD Small versus Large	0.391	0.222	0.686	10.7068	**.0011**	0.4690	0.343	0.641	22.5951	**<.0001**
OWNGRPCD				10.8443	**.0126**				11.9482	**.0076**
OWNGRPCD forest service versus state and local	0.406	0.117	1.405	2.0261	.1546	0.536	0.183	1.574	1.2882	.2564
OWNGRPCD Other federal versus State and Local	0.866	0.198	3.792	0.0366	.8483	1.135	0.380	3.394	0.0517	.8201
OWNGRPCD Private versus State and Local	1.692	1.063	2.693	4.9199	**.0265**	1.717	1.137	2.595	6.6009	**.0102**
ECOSECT				233.0264	**<.0001**				398.0507	**<.0001**
ECOSECT 211E versus 221B	3.04	0.967	9.554	3.6291	.057	1.095	0.497	2.414	0.0509	.8214
ECOSECT 211F versus 221B	0.47	0.143	1.549	1.5394	.2147	0.307	0.158	0.597	12.1075	**.0005**
ECOSECT 212K versus 221B	3.083	1.051	9.041	4.2064	**.0403**	0.579	0.300	1.117	2.6537	.1033
ECOSECT 212Q versus 221B	2.649	0.890	7.883	3.0633	.0801	0.596	0.290	1.226	1.9779	.1596
ECOSECT 212T versus 221B	0.195	0.035	1.078	3.5129	.0609	0.304	0.151	0.611	11.1588	**.0008**
ECOSECT 212X versus 221B	0.615	0.196	1.93	0.694	.4048	0.187	0.091	0.384	20.7921	**<.0001**
ECOSECT 221E versus 221B	0.369	0.120	1.136	3.0202	.0822	0.203	0.109	0.376	25.6067	**<.0001**
ECOSECT 222H versus 221B	0.634	0.191	2.104	0.5553	.4562	0.462	0.203	1.053	3.3741	.0662
ECOSECT 222I versus 221B	2.146	0.701	6.567	1.7891	.181	0.499	0.240	1.034	3.5004	.0614
ECOSECT 222J versus 221B	0.19	0.035	1.049	3.628	.0568	0.564	0.292	1.091	2.8973	.0887
ECOSECT 222L versus 221B	4.108	1.486	11.358	7.4169	**.0065**	2.068	1.204	3.552	6.9229	**.0085**
ECOSECT 222M versus 221B	2.51	0.849	7.424	2.7658	.0963	0.498	0.248	1.000	3.8371	.0501
ECOSECT 223A versus 221B	0.403	0.123	1.326	2.2366	.1348	0.122	0.062	0.242	36.562	**<.0001**
ECOSECT 223E versus 221B	0.339	0.098	1.176	2.9069	.0882	0.314	0.089	1.106	3.251	.0714
ECOSECT 251C versus 221B	0.186	0.041	0.842	4.7647	**.029**	0.206	0.103	0.412	19.8511	**<.0001**
ECOSECT M221A versus 221B	0.633	0.188	2.133	0.5446	.4605	0.356	0.182	0.695	9.1618	**.0025**
ECOSECT M221B versus 221B	0.435	0.115	1.649	1.4995	.2208	0.298	0.147	0.604	11.2558	**.0008**
ECOSECT M221C versus 221B	0.754	0.229	2.489	0.2139	.6437	0.355	0.178	0.710	8.5766	**.0034**
PHYSCLCD				6.674	**.0355**				2.6006	.2725
PHYSCLCD Mesic versus Xeric	2.041	0.979	4.256	3.6256	.0569	1.518	0.207	11.151	0.1682	.6817
PHYSCLCD Hydric versus Xeric	0.562	0.112	2.829	0.4891	.4843	0.312	0.019	5.195	0.6599	.4166
FORTYPGRP				44.6087	**<.0001**				45.6444	**<.0001**
FORTYPGRP White/Red/Jack Pine versus Oak/Pine	0.266	0.048	1.468	2.3094	.1286	1.588	0.331	7.615	0.3347	.5629
FORTYPGRP Loblolly/Shortleaf Pine versus Oak/Pine	0.993	0.108	9.114	0	.995	–	–	–	–	–
FORTYPGRP Oak/Hickory versus Oak/Pine	2.109	0.770	5.774	2.1079	.1465	4.464	1.097	18.161	4.3653	**.0367**
FORTYPGRP Elm/Ash/Cottonwood versus Oak/Pine	1.687	0.575	4.955	0.906	.3412	3.954	0.932	16.777	3.4762	.0623
FORTYPGRP Maple/Beech/Birch versus Oak/Pine	3.714	1.340	10.292	6.3657	**.0116**	6.082	1.486	24.889	6.3062	**.012**
FORTYPGRP Aspen/Birch versus Oak/Pine	0.745	0.234	2.368	0.2487	.618	1.619	0.367	7.146	0.4045	.5248
FORTYPGRP other hardwoods versus Oak/Pine	1.777	0.316	9.992	0.4259	.514	–	–	–	–	–

STAGE is stand age, STDSZCD is stand‐size class, OWNGRPCD is ownership group, ECOSECT is ecoregion section, PHYSCLCD is physiographic class, and FORTYPGRP is forest‐type group.

Bold indicates significance (*p* <0* *.05).

**Table 6:** Performance of the logistic regression model of butternut occurrence by time period based on receiver operating characteristic (ROC) curve area, max‐rescaled *r*‐square value, and chi‐square values for the parameter estimates in Table 5

Time period	Sample size (*n*)	ROC curve area	Max‐Rescaled	Chi‐square statistic					
			*R*‐square	Variable					
				OWNGRPCD	STAGE	STDSZCD	ECOSECT	FORTYPGRP	PHYSCL
2	23,440	0.810	0.1398	11	8	15	233	45	7
1	21,119	0.783	0.1346	12	2	28	398	46	3

OWNGRPCD is ownership group, STAGE is stand age, STDSZCD is stand‐size class, ECOSECT is ecoregion section, FORTYPGRP is forest‐type group, and PHYSCL is physiographic class.

Comparison of the odds ratios from both models indicated that butternut occurrence was significantly higher than the reference section, 221B, in ecoregion section 222L (Table 5), North‐Central U.S. Driftless and Escarpment, which is characterized as an unglaciated upland plateau with steep‐sided bedrock ridges and mounds (McNab et al., 2007). The odds ratios for the time 2 model also indicated that butternut occurrence was significantly higher than the reference section in ecosection 212K (Table 5), Western Superior Highlands, which is characterized by uniform, undulating, poorly drained, level to rolling landscape of glacial drift plains (McNab et al., 2007). Similarly, odds ratios from both models indicated that butternut occurrence was significantly higher than the reference forest‐type group, oak/pine, in the maple/beech/birch group and that occurrence was significantly higher on private lands than the reference ownership group, state and local governments. Finally, odds ratios from both models indicated that butternut occurrence was significantly lower than that of the reference stand size, large‐diameter stands, in small‐diameter stands, while odds ratios from the time 1 model indicated that occurrence was significantly lower in medium‐diameter stands.

## 4 DISCUSSION

Although butternut continues to be present across much of the range described by Little (1971; Figure 1), the abundance and volume of butternut trees have decreased across this range and in nearly all states (Table 1). It occurs at the highest densities in ecoregion sections 222L, 221H, 221E, and 222I (Figure 1), but the largest declines have also occurred in some of these sections, particularly 222L (Tables 1 and 3; Figures 3 and 4). Despite the apparent decrease in butternut tree abundance across its range and in most states, butternut volume has increased in 211E, 212K, 221H, and 222I although the increases were not statistically significant (Tables 2 and 3). Substantial decreases in the populations of butternut trees across nearly all diameter classes have occurred (Figure 2), but the loss of trees from the largest diameter classes has comparably larger ecological and economic consequences including reduced availability of mast, seed trees, den trees, carbon stores, and lumber.

The spatial variation in changes in butternut tree abundance and volume and annual mortality rates (Figures 3 and 4; Table 3) may indicate differences in the virulence of the fungus causing butternut canker, the presence of disease‐resistant butternut trees, or ecological site differences in the local environment that contribute to tree survival. Broders, Boraks, Barbison, Brown, and Boland (2015) reported that two different strains of *O. clavigignenti‐juglandacearum* may have been introduced in North America: a more virulent strain in Minnesota and Wisconsin in the 1960s and a less virulent strain that may have been present in the northeastern United States for far longer. The relative and annual decreases in numbers and volume reported here may reflect the difference between these two strains; the largest decreases in numbers and volume were concentrated in the Midwest and Lake States (Figures 3 and 4), but increases in butternut volume were concentrated in the 211E, 221H, and 223E (Figure 4c,d).

Although there may be some variation in the heritability of resistance, there are no studies demonstrating heritable resistance to butternut canker disease. One study demonstrated little genetic basis for observed differences in mortality (LaBonte, Ostry, Ross‐Davis, & Woeste, 2015). Instead, LaBonte et al. (2015) concluded that localized site conditions were more useful predictors of butternut mortality with greater survival in drier, upland sites.

Our results indicate that ecoregion section was by far the most important environmental variable for predicting butternut occurrence (Table 6). This variable accounts for many different regional characteristics including the tendency to occur in upland regions. Forest‐type group and stand‐size class were also important predictors of occurrence. Occurrence of surviving butternut was significantly greater in large‐diameter stands which could indicate a lack of regeneration opportunities. Not only are surviving butternut lacking in small‐ and medium‐diameter stands, but successful regeneration of shade‐intolerant butternut is unlikely in large‐diameter stands absent disturbance. Indeed, a study by Parks, Jenkins, Woeste, and Ostry (2013) reported that butternut regeneration in the Great Smoky Mountains National Park was continuous from the 1930s to around 1980 and then declined drastically. Ownership group, stand age, and physiographic class, while significant in one or both models, were far less important. The logistic regressions indicated that restoration efforts should be focused in large‐diameter maple/beech/birch stands in sections 222L and 212K due to the relatively high number of butternut that remain there and 222I and 211E due to the stability of the butternut populations.

Although canker‐resistant trees have not yet been developed, the trees that are still surviving in stands that have been impacted by butternut canker may have greater resistance to the disease due to variability in host genetics, environmental conditions, pathogen genetics, or a combination of these factors. These sections are located in eastern Minnesota and western Wisconsin (Figure 1). Furthermore, based on other literature on butternut canker impacts (LaBonte et al., 2015), restoration of butternut is likely to be most successful on dry, upland sites within forest stands with the aforementioned ecological characteristics. The stands with surviving butternut also could be used to study butternut regeneration and disease epidemiology. One complication for restoration if hybrid butternut genotypes are employed is that optimal habitats for restoration may be different from habitats where butternut has historically occurred. Hybrids may be best suited for planting at a different set of sites than would be appropriate for restoration using pure butternut genotypes (Crystal & Jacobs, 2014; Crystal, Lichti, Woeste, & Jacobs, 2016).

We report here a ca. 43% decline in numbers of butternut trees and 58% decline in butternut volume across the species range during a 30‐ to 40‐year period. Although this is a large decline, it is substantially less than what has been reported elsewhere (U.S. Forest Service, 2005). During this same time period, the volume of black walnut (*J. nigra* L.), a species with a similar geographic distribution and shade tolerance classification, has increased by 125 percent. While we do not have any direct evidence of the cause of this change, the decline is most likely a result of tree mortality caused by the fungal pathogen *O. clavigignenti‐juglandacearum* that may have been introduced from Asia many decades ago (Furnier et al., 1999; Nair, 1998; Ostry, 1998b; Ostry et al., 1994) or was a native fungal endophyte or minor pathogen that made a host jump to butternut (Broders, Boraks, Sanchez, & Boland, 2012). This conclusion is supported by focused surveys indicating infection by this pathogen as the major cause of butternut mortality (U.S. Forest Service, 2005).

The declining presence of butternut in North America thus represents another example of regional changes in forest composition driven by invasions of non‐native insects and pathogens (Lovett et al., 2016), a problem that is particularly acute in eastern N. America (Liebhold et al., 2013). Other examples of massive declines of North American tree species caused by insect and pathogen invasions include the demise of American chestnut, *Castanea dentata*, caused by the exotic fungal pathogen *Cryphonectria parasitica* (Dalgleish, Nelson, Scrivani, & Jacobs, 2016), the regional decline in American beech, *F. grandifolia*, caused by beech bark disease (Morin & Liebhold, 2015) and the current wave of ash, *Fraxinus* spp., mortality caused by the emerald ash borer (Morin, Liebhold, Pugh, & Crocker, 2016). While the regional decline in abundance of butternut documented here is similar to these other examples in its regional scale, it is unique in that butternut was not a common tree, even before the invasion of the butternut canker pathogen. From a conservation perspective, the impacts of butternut canker are thus particularly acute as the pathogen invasion pushes a rare tree species toward extinction, at least at a local scale. Despite the rarity of butternut, its unique ecological characteristics including large mast may have a disproportionate impact on wildlife populations. Butternut restoration offers a chance to reverse this trend, particularly if efforts are focused in forest stands most suitable for tree growth and recruitment.

## CONFLICT OF INTEREST

None declared.
