# Supplementary material for: Regional patterns of declining butternut (Juglans cinerea L.) suggest site characteristics for restoration
Source: Ecol Evol. 2017 Dec 1;8(1):546–59. doi: 10.1002/ece3.3641 (PMC5756827; doi:10.1002/ece3.3641)
Supplement: Supplementary file 1 [file ECE3-8-546-s001.docx]

| Appendix S1.--Years of periodic and annual FIA inventory data and time interval between surveys by state. | | | |
| --- | --- | --- | --- |
|  |  |  |  |
|  | Time 1 | Time 2 |  |
| State | periodic | annual | Interval |
| Alabama | 1982 | 2015 | 33 |
| Arkansas | 1978 | 2015 | 37 |
| Connecticut | 1985 | 2015 | 30 |
| Illinois | 1985 | 2015 | 30 |
| Indiana | 1986 | 2015 | 29 |
| Iowa | 1990 | 2015 | 25 |
| Kentucky | 1988 | 2013 | 25 |
| Maine | 1995 | 2015 | 20 |
| Massachussetts | 1985 | 2015 | 30 |
| Michigan | 1980 | 2015 | 35 |
| Minnesota | 1977 | 2015 | 38 |
| Missouri | 1989 | 2015 | 26 |
| New Hampshire | 1983 | 2015 | 32 |
| New Jersey | 1987 | 2014 | 27 |
| New York | 1993 | 2014 | 21 |
| Ohio | 1991 | 2014 | 23 |
| Pennsylvania | 1989 | 2014 | 25 |
| Tennessee | 1980 | 2013 | 33 |
| Vermont | 1983 | 2015 | 32 |
| West Virginia | 1989 | 2014 | 25 |
| Wisconsin | 1983 | 2015 | 32 |
