# Supplementary material for: Regional patterns of declining butternut (Juglans cinerea L.) suggest site characteristics for restoration
Source: Ecol Evol. 2017 Dec 1;8(1):546–59. doi: 10.1002/ece3.3641 (PMC5756827; doi:10.1002/ece3.3641)
Supplement: Supplementary file 2 [file ECE3-8-546-s002.docx]

| Appendix S2.--States included and average interval between surveys by ecoregion section. | | |
| --- | --- | --- |
|  |  |  |
| Ecoregion Section | States | Average Interval |
| 211E | NY, VT | 26.5 |
| 211F | PA, NY | 23.0 |
| 212K | MN, WI | 35.0 |
| 212Q | MN, WI | 35.0 |
| 212T | MI, WI | 33.5 |
| 212X | MI, WI | 33.5 |
| 221B | NJ, NY, PA, VT | 26.3 |
| 221E | KY, OH, PA, WV | 24.5 |
| 221H | KY, TN | 29.0 |
| 222I | NY, OH, PA | 23.0 |
| 222J | IL, IN, MI, OH | 29.3 |
| 222L | IL, IA, MN, WI | 31.3 |
| 222M | IA, MN, WI | 31.7 |
| 223A | AR, IL, MO | 31.0 |
| 223E | AL, KY, TN | 30.3 |
| 251C | IL, IA, MO | 27.0 |
| M221A | ME, NH, VT | 28.0 |
| M221B | MA, NH, VT | 31.3 |
| M221C | CT, MA, NY, VT | 28.3 |
|  | All States | 29.0 |
